# Social, Digital and Community Capital Facilitated COVID-19 Pandemic Resilience in a Qualitative Survey of Older Adults With Mild Cognitive Concerns

**DOI:** 10.1177/00469580251332062

**Published:** 2025-06-13

**Authors:** Annie Mae Wright, Harriet Demnitz-King, Alexandra Burton, Rachel M. Morse, Sweedal Alberts, Charlotte Kenten, Rosario Isabel Espinoza Jeraldo, Michaela Poppe, Julie Barber, Claudia Cooper

**Affiliations:** 1 Queen Mary University of London, UK; 2 University College London, UK

**Keywords:** mild cognitive impairment, prevention, COVID-19, lifestyle

## Abstract

The COVID-19 pandemic negatively affected known dementia risk factors and cognition in older adults. Our objective was to explore how older adults with cognitive concerns (ie, MCI or SCD) were able, or not able to engage in lifestyle activities associated with dementia prevention and maintain their wellbeing. We invited adults with mild cognitive concerns without dementia, aged ≥60 years participating in a randomised controlled trial of a psychosocial, secondary dementia prevention intervention, to complete a semi-structured survey, regarding how the pandemic impacted their lifestyle and wellbeing in areas relevant to dementia risk: social connections, activities, diet, mental and physical health, community and family support. Data was collected between October 2020 and December 2022; we inductively coded responses using manifest content analysis. 551/748 trial participants completed the survey. Most (n = 530, 96%) described pandemic-related lifestyle or wellbeing changes; two thirds (n = 369/545, 67.7%) reported less activities. A quarter (n = 145, 26.8%) identified no change in social connections, with others reporting less in-person meetings (n = 139, 25.7%) or speaking to less people (n = 99; 18.2%); a minority engaged in compensatory online activities (n = 31, 5.7%) and online (n = 63, 11.6%) or phone (n = 90, 16.6%) social contact. Relatively few reported weight gain (n = 22, 4.0%); two-thirds reported no change in their diet (n = 360, 66.1%). Modes of support changed, with reliance on food parcels, online services and shopping by neighbours. Over half reported (almost exclusively negative) mental health pandemic-related changes (n = 307, 56.9%), including depression, stress, fear and loneliness; many reported declines in physical health (n = 153, 28.1%) and/or fitness (n = 70, 12.8%). Stoical accounts of adaptation and resilience, enabled by technology and community support predominated, but were not possible for all. Creating communities where cognitively frail people are more digitally and socially connected will support resilience of this group and contribute to dementia prevention, now and in any future pandemic.

Trial registration- ISRCTN17325135

**Highlights**

● The pandemic was a particularly challenging time for people with mild cognitive concerns without dementia: a group relatively overlooked by current and contemporary policy.

● Renegotiating daily routines was facilitated by support of community for most participants.

● Preparedness planning for future national disasters should consider the needs of people with mild cognitive, in whom support to continue lifestyle and wellbeing routines that reduce dementia risk can enable adaptation during crises and subsequent cognitive wellbeing.

## Introduction

A quarter of the UK population aged 60 and over, live with cognitive impairments that are not dementia but infer an increased risk of dementia.^1,2^ People meeting criteria for these risk categories are sometimes described as having Mild Cognitive Impairment (MCI), when there are objective cognitive deficits, or Subjective Cognitive Decline (SCD), when deficits are only subjectively measurable. The Lancet Commission on Dementia Prevention, Intervention and Care 2024 identified 14 modifiable risk factors with sufficient evidence – less education, hearing, vision loss, high LDL cholesterol, depression, traumatic brain injury, physical inactivity, diabetes, smoking, hypertension, obesity, excessive alcohol consumption, social isolation and air pollution – that, if addressed, might potentially prevent up to 45% of dementia cases worldwide.^3^ These factors are also relevant to those experiencing MCI and SCD, as secondary prevention strategies.^4^

Globally, government measures to limit the spread of Covid-19 through social distancing, lockdowns, quarantine and stay at home orders saved lives,^5^ but the ensuing isolation and anxieties harmed health and wellbeing. People living with dementia were particularly affected; social isolation worsened neuropsychiatric and behavioural symptoms^6^ and accelerated functional^6^ and cognitive decline.^7^ Fear of infection, isolation and closure of dementia services severely affected their wellbeing and that of their family carers.^8^ The COVID-19 pandemic resulted in a deleterious effect on known dementia risk factors (eg, loneliness, substance misuse, reduced exercise).^9^ People with MCI and SCD reported more detrimental lifestyle changes, including reduced physical activity, increased smoking and greater alcohol consumption, than people without cognitive impairments,^10^ as well as increased rates of frailty and depression.^11^

The National Risk Register emphasises the importance of learning from the pandemic, so that planning for future events is based on a broad understanding of potential health, social, financial and environmental impacts and community capacity and capabilities to support preparedness, response and recovery, in particular for vulnerable groups.^12^ Resilience, defined as the ability to adapt well in the face of difficulties, has been proposed as a defence against loneliness and social isolation during the pandemic and was probably an important buffer between this and how other pandemic-related stressors affected lives.^13^ Personal resources (eg, psychological resilience, self-efficacy) and social resources (eg, emotional support, social connectedness) can mitigate threats to physical and mental health, social adjustment and quality of life.^13^ One study found that participation in physical exercise during the pandemic reduced anxiety in older adults, while social participation supported mental resilience.^14^ Several studies have reported how technology use, specifically video-calling, buffered pandemic-induced loneliness and isolation.^13^

The ability to build resilience is an interaction between individuals and the social environment and should not be construed as an individual achievement.^15^ Not all older people are equally able to exhibit resilience, leading to new social divisions. For example, those living with mild cognitive concerns may be less able to make changes to reduce dementia risk. An emphasis on agency burdens individuals with the personal responsibility of staying healthy, whether or not this is possible; dementia prevention is a societal concern.^16^

In, to our knowledge, the largest study on how the pandemic affected the lifestyle and wellbeing of people with mild cognitive concerns to date, we drew on the conceptual framework of resilience^17^ to consider how older people experiencing cognitive concerns were able or not to maintain healthy lifestyle behaviours and connections in the dual challenging contexts of cognitive impairments and pandemic-related social restrictions. We recruited participants from a Randomised Controlled Trial (RCT) of a dementia prevention intervention, APPLE-Tree (Active Prevention in People at risk of dementia through Lifestyle, bEhaviour change and Technology to build REsiliEnce).^18^ We aimed to explore how older adults with cognitive concerns (ie, MCI or SCD) were able, or not able to engage in lifestyle activities associated with dementia prevention and maintain their wellbeing. We considered what accounts of resilience (adaptation of routines to pandemic contexts) or challenges to adapting, might tell us about how resilience is best supported and maintained in this population in adverse situations.

## Method

We followed Consolidated criteria for reporting qualitative research (COREQ) (Appendix 2).^19^ We conducted our study in accordance with the Helsinki Declaration of 1975 as revised in 2024.

### Study Sample

All participants recruited for the APPLE-Tree RCT investigating the effectiveness of a multidomain dementia prevention intervention on reducing cognitive decline in people with cognitive concerns^18^ were invited to complete the semi-structured, qualitative survey during the baseline assessment. The APPLE-Tree Trial recruitment took place in participating primary care practices and secondary care memory services; in these settings, which accounted for the majority of recruitment, all eligible participants were approached by letter, targeted at those with some markers of frailty, inviting them to contact researchers if they were worried about their memory, or approached directly by NHS staff (memory services). We also recruited through charities for older people: the Joint Dementia Research Register and social media, newspaper and online advertisements.

We included people aged 60+, who met criteria for MCI or SCD. This was operationalised as either a Quick MCI test score^20^ between 50 and 61 (participants scoring <50 were included if their low scores were consistent with MCI/SCD due to, for example, educational attainment or speaking English as a second language) or, alternatively, a score of ≥62 with a ‘yes’ response to the question, ‘Has your memory deteriorated in the last 5 years? Or has a friend/family member noticed it deteriorating?’ and at least one of the following questions: ‘Is your memory persistently bad, or has a friend/family member noticed it being persistently bad?’ or ‘Are you or others around you concerned about this?’. We adapted this approach from published measures of SCD.^21,22^ Further inclusion criteria were a Functional Assessment Questionnaire score <9 indicating no significant cognitive impairment^23^ and having a family member/friend/professional to act as an informant who was in contact with the participant at least once a month. An additional inclusion criterion was a willingness to engage in a videocall group intervention for the APPLE-Tree study.

We excluded people with a diagnosis of a primary neurodegenerative disease, advanced, severe unstable or terminal medical condition or severe mental illness or who lacked capacity to consent. We also excluded people with an AUDIT-C (Alcohol Use Disorders Identification Tool) score ≥8, indicative of harmful alcohol use.^24^ Lack of access to WIFI or a device to access video calls was not an exclusion criterion as participants were given assistance to access video calls and provided with Mifi and tablets to use throughout the intervention, if required. For participants without online access, the covid questionnaire details that are described in this paper were collected in person or on the phone. Full details of the APPLE-Tree trial, including detailed eligibility criteria, are published (27).

### Procedure

Almost all assessments took place by video-call, but when it facilitated participation, a small number were conducted face-to-face where COVID restrictions allowed. We followed government guidance on social distancing during face-to-face interviews.^25^ In line with procedures approved by the ethics committee, trained researchers obtained written (or audio-recorded) informed consent from participants and informants. Each participant was asked to provide information on their age, gender, ethnicity and any diagnoses related to memory. As part of the baseline assessment, fully detailed in the main protocol (27), questions were developed in consultation with the APPLE-Tree Patient and Public Involvement group to ask about changes to lifestyle and wellbeing which they attributed to the pandemic. A PPI member piloted the questionnaire. They used as a starting point the lifestyle and wellbeing changes identified as modifiable risk factors for dementia, that were the focus of the APPLE-Tree intervention^26^: healthy eating, increased social connections, physical and mental activity and looking after mental and physical health. Respondents were asked about ‘recent changes due to COVID-19’; the researcher asked them to compare their current situation to pre-pandemic. The questions asked were: “How did the recent changes due to COVID-19 change:

a. Who you speak to each week?
b. What you eat?
c. What activities you do?
d. How you access help and who you turn to if you need:
  ● emotional support?
  ● practical help?
e. Your mental wellbeing (eg, worries, mood)?
f. Your physical wellbeing?
g. Who you provide care for?”

This was an unvalidated questionnaire developed by the co-authors (Supplemental File 1). Responses were free-text; then coded by researchers (see analysis section). Participants could decide to self-complete the questionnaire having been sent this via post or email after meeting with the researcher for the baseline APPLE-Tree interview, or to complete with the researcher, who also recorded notes including verbatim quotes. Thus most quotes in the results section are in participants’ own words, while a small number are in the third person, where the participant opted to reply to questions verbally while the researcher made notes. Unless the participant opted to complete the questionnaire with a friend or family member present, only the researcher and participant were present during interviews. The APPLE-Tree assessment took around 90 minutes, and the COVID questionnaire 5 to 10 minutes. Interviews were not audio/video recorded, and no additional fieldnotes were taken.

Researchers were from non-clinical, graduate health or sociology backgrounds, most were female and they were employed at a university to work on the trial and trained in data collection and delivery of the intervention.^27^ They typically contacted participants before baseline assessments to plan a suitable date or time, but otherwise were not previously known to them. At the start of assessments, they introduced their role, to collect data prior to randomisation to study group, for the purpose of understanding how the pandemic was affecting daily lives. AMW (lead researcher) was a medical student who undertook this study for her dissertation, supervised by CC and RM. SA and RJE were study researchers, CC (academic psychiatrist) was Chief Investigator and AB (applied mental health researcher) a co-investigator. CK and HDK were postdoctoral researchers with geography and psychology backgrounds respectively.

### Data Analysis

We used manifest content analysis, which involves creating codes directly from the recorded text to analyse the broad surface structure of the data.^28^ We used Nvivo software to organise data. We generated codes inductively, applying the 4 stages of content analysis ^29^: decontextualisation, recontextualisation, categorisation and compilation. The researcher AMW conducted the analysis and CC independently coded 10% of responses; the level of agreement was ≥90%. We used the broad categories outlined in our questions (a-g above), inductively coding content within these to develop our coding tree (Appendix 1). In interpreting data, we considered how our findings were situated within our selected conceptual framework of resilience, considering how personal resources, including resilience and social resources influence coping.^17^

## Results

### Sample Description

Surveys were completed between October 2020 and December 2022. Of 748 APPLE-Tree trial participants, 551 (74%) completed the COVID-19 questionnaire (numbers responding to each question shown in Table 2). Reasons for non-completion were not formally reported, but were typically due to questionnaire fatigue as the survey comprised the final part of the assessment battery. Sociodemographic characteristics of those completing questionnaires were comparable to the baseline trial sample (Table 1). Of the individuals who completed the questionnaire, 132 (24%) did so between October 2020 and July 2021 when a national lockdown was in place, while 419 (76%) participants responded after legal restrictions were removed (August 2021-December 2022) (3). During this second period, there were ongoing social restrictions, for example, face masks were compulsory in public spaces until the end of January 2022. We considered during analysis how timing of the interview might influence data. Most participants (n = 512/551, 93%) had access to online video-calling during the pandemic. As shown in Table 1, while all participants met trial criteria for MCI or SCD (see inclusion criteria), not all self-identified as having conditions with these labels at the time of interview; some when asked whether they had received a diagnosis for their cognitive concerns of MCI responded that they had, others identified as having memory concerns or problems, having no diagnosis or label for their memory concerns or gave other explanations around their symptoms. Content analysis findings (Table 2).

**Table 1.** Baseline Characteristics of APPLE-Tree Trial Participants and Those Participating in the Qualitative Survey.

Variable	Trial population (n = 745)*	Survey population (n = 551)
Age – mean (SD) range	74.3 (6.9) (n = 744) 57.9-102.7	74.4 (7.0) (n = 551) 60.05-102.74
Gender	745	n = 551
Male	394 (52.9%)	287 (52.1%)
Female	350 (47.0%)	263 (47.7%)
Other	1 (0.1%)	1 (0.2%)
Ethnicity	744	n = 551
White UK	601 (80.8%)	442 (80.2%)
White other	57 (7.7%)	47 (8.5%)
Mixed	16 (2.2%)	11 (2.0%)
Asian	50 (6.7%)	38 (6.9%)
Black	13 (1.7%)	9 (1.6%)
Other	7 (0.9%)	4 (0.7%)
Marital status	744	n = 551
Single	46 (6.2%)	32 (5.8%)
Married/civil partnership	464 (62.4%)	342 (62.1%)
Living with partner	30 (4.0%)	23 (4.2%)
Widowed	108 (14.5%)	78 (14.2%)
Divorced	94 (12.6%)	74 (13.4%)
Unable to specify	2 (0.3%)	2 (0.4%)
Highest level of education	744	n = 551
No education	2 (0.3%)	1 (0.2%)
Primary	10 (1.3%)	8 (1.5%)
Secondary (eg, O level; GCSE)	167 (22.4%)	130 (23.6%)
Further (eg, A level; BTEC; NVQ)	201 (27.0%)	142 (25.8%)
Degree	204 (27.4%)	156 (28.3%)
Postgraduate	147 (19.8%)	106 (19.2%)
Other	11 (1.5%)	6 (1.1%)
Unable to specify	2 (0.3%)	2 (0.4%)
Employment	744	n = 551
Full time employment	32 (4.3%)	19 (3.4%)
Part time employment	54 (7.3%)	40 (7.3%)
Retired	608 (81.7%)	450 (81.7%)
Unemployed/unable to work	18 (2.4%)	14 (2.5%)
Other	29 (3.9%)	26 (4.7%)
Unable to specify	3 (0.4%)	2 (0.4%)
Living situation	744	n = 551
Live alone	200 (26.9%)	147 (26.7%)
Live with other relatives	532 (71.5%)	393 (71.3%)
Live with friends/other people	6 (0.8%)	5 (0.9%)
Other	6 (0.8%)	6 (1.1%)
Type of accommodation	744	n = 551
Council rented	33 (4.4%)	26 (4.7%)
Private rented	40 (5.4%)	25 (4.5%)
Own home	655 (88.0%)	485 (88.0%)
Supported living	12 (1.6%)	11 (2.0%)
Other	4 (0.5%)	4 (0.7%)
Diagnosis	744	n = 551
Mild cognitive impairment	125 (16.8%)	94 (17.1%)
Memory Concerns or problems	59 (7.9%)	38 (6.9%)
Other	13 (1.7%)	11 (2.0%)
Not given diagnosis	527 (70.8%)	394 (71.5%)
Unable to specify	20 (2.7%)	14 (2.5%)

* Of the 748 participants who completed baseline assessments and were randomised, demographic data were missing for 3 participants.

**Table 2.** Description of Content Analysis Findings.

Code	% (n)*	Example quote
How did the pandemic change who you speak to each week? (n = 541)		
No change	26.8 (n = 145)	Not much change
Less in-person	25.7 (n = 139)	I now speak to people and attend activities over Zoom rather than in person
Less people	18.3 (n = 99)	I see fewer people. No parties, people can’t come over for meals.
More phone calling	16.6 (n = 90)	I phone friends now
More online calling	11.6 (n = 63)	Yes, I use facetime quite a lot.
More people	4.6 (n = 25)	Covid increased it. I am phoning friends and family more.
How did the recent changes due to COVID change what activities you do? (n = 545)		
No change	15.4 (n = 84)	No difference
Less activities	67.7 (n = 369)	Yes, much less activities than I used to do.
Less exercise	22.4 (n = 122)	I used to do a lot of swimming and swam at ponds. I’m no longer going out and about.
Less travel or holidays	5.5 (n = 30)	We did have some big trips booked which have been cancelled.
Less theatre outings	4.6 (n = 25)	I have not been able to go to the theatre or cinema, or to see live music.
Less gym sessions	4.6 (n = 25)	I used to go to the gym 3 times a week and I can’t now.
Less in-person shopping	4.4 (n = 24)	I haven’t been able to go out shopping.
More walking	16.0 (n = 87)	I deliberately was going out for a walk, I was trying to walk more.
More online activities	5.7 (n = 31)	Not going to the ‘centre’ now, its all over Zoom.
More indoor activities	4.8 (n = 26)	I was staying inside and playing board games with my brother.
More gardening	3.1 (n = 17)	I did more gardening too.
How did the recent changes due to COVID change your physical wellbeing? (n = 545)		
No change	50.5 (n = 275)	No change.
Decline in physical health	28.1 (n = 153)	It slowed me down, I wasn’t exercising as much.
Reduced fitness or exercise	12.8 (n = 70)	Haven’t been doing the sort of exercise I used to do. I used to be out and about a lot.
Weight gain	4.0 (n = 22)	I put on a bit of weight because I’m not as active.
More aches and pains	2.0 (n = 11)	I feel more stiff, more pains.
Reduced access to healthcare services	1.5 (n = 8)	Would like to see my GP, consultant etc. which I’m not doing at the moment.
Improvement in physical health	8.3 (n = 45)	Strangely enough I’ve lost weight, about a stone, because I haven’t been going down to the pub.
Increased fitness or exercise	6.4 (n = 35)	I improved because I exercise more outside.
How did the recent changes due to Covid change what you eat? (n = 545)		
No change	66.1 (n = 360)	Same as before
More cooking at home/less eating out	10.5 (n = 57)	I have increased home cooking, and have a takeaway once per week. So I definitely cook more at home now.
More unhealthy food	5.7 (n = 31)	I eat more chocolate at night as a treat - I don’t usually do this.
More healthy eating	4.8 (n = 26)	More positive actually, I protect myself by eating more healthily
More food	3.9 (n = 21)	Early days I was eating a lot, we were cooking and my daughter was baking all the time
More snacking	2.0 (n = 11)	I’m probably snacking more than I used to
How did the recent changes due to COVID change your mental wellbeing? (n = 540)		
No change	43.1 (n = 233)	No change, I have plenty of support
Lower mood	40.7 (n = 220)	Low mood due to lockdown - not seeing friends and family.
More worry or stress	12.0 (n = 65)	Yes, increased worrying affecting my mood.
More anxiety	6.1 (n = 33)	I panic all the time and feel anxious all the time and I don’t like that. I didn’t have it before.
More loneliness	4.1 (n = 22)	I feel more loneliness due to isolation
Fear of the virus	4.4 (n = 24)	Yes - he was scared of catching COVID and ending up in hospital and dying from COVID
Described feeling depressed	4.1 (n = 22)	I got very depressed as I was stuck in my room.
Better mood	2.4 (n = 13)	I sleep a lot more so it’s been good for my mental health.
How did the recent changes due to COVID change how you access help and who you turn to if you need practical support? (n = 546)		
No change	56.4 (n = 308)	People who live nearby are still available
Less access to practical support	10.4 (n = 57)	Son couldn’t come round to help around the house
Needed and/or received more practical support	9.9 (n = 54)	I’ve required more practical help during COVID
Less access to cleaners/tradesmen/gardeners	4.2 (n = 23)	Very much, I can’t have contractors, cleaners etc. over because I am diabetic I have to be shielding.
Change to accessing medical services	1.6 (n = 9)	I visit an online surgery, not seeing the GP face-to-face as much
How did the recent changes due to COVID change how you access help and who you turn to if you need emotional support? (n = 540)		
No change	66.5 (n = 359)	Hasn’t changed, has always been my husband
Had less access to emotional support	7.8 (n = 42)	I feel like I am struggling to get to the doctors, I am struggling to get help.
Change to how they accessed emotional support	6.5 (n = 35)	I now gain emotional support via telephone
Needed and/or received more emotional support	3.1 (n = 17)	I was referred to Talking Therapies because I was panicking a lot about travelling
How did the recent changes due to COVID change who you provide care for? (n = 538)		
No change/do not provide care	66.9 (n = 360)	I don’t provide care for anyone
Provided more care for others	17.7 (n = 95)	The old lady below us - I’m more conscious to cook food for her to take down
Provided more emotional support for others	2.4 (n = 13)	I provide emotional support to friends over the phone
Brought shopping or provisions to others	3.0 (n = 16)	I bring groceries to my neighbour, so I provide care occasionally.
Unable to provide as much support for others	11.0 (n = 59)	Yes I have a daughter in a care home so haven’t seen her and haven’t been able to provide care for her.

* Note that responses could be coded in no, one or more than one category.

530/551 (96%) of participants described experiencing some change to their lifestyle or wellbeing because of the pandemic, while 21 (4%) described no changes; of these, 3 responses were collected before July 2021 and eighteen after.^30^

Content analysis findings are detailed in Table 2, with example quotes, and discussed below. In summary, most participants identified a decrease in opportunities for social connections and activities, balanced for some by greater use of video-call and phone, and online groups. Changes to diet were less frequent, as were changes to support received or given. Just under half of participants reported decline in their mental and physical wellbeing, and many linked this to the reduced social and activity opportunities during the pandemic, Note that categories reported for each question were not mutually exclusive, respondents could endorse more than one. The coding tree is included in Appendix 1.

### Changes to Who Participants Spoke to (n = 541)

A quarter (n = 145, 26.8%) identified no change in social connections, with others reporting less in-person meetings (n = 139, 25.7%) or speaking to less people (n = 99; 18.3%). The following was a typical response, describing the impact of fewer social contacts:

*‘There was a big change, I speak to less people every week. I am also speaking to less people out and about’* (female, aged 60-64, responded before August 2021).

This was to some extent balanced with more telephone communication (n = 90, 16.6%) and more video-call contact (n = 63, 11.6%). For a minority, (n = 25, 4.6%) the focus on remote (phone or video-call) connections enabled more social connections than pre-pandemic.

*‘I speak often to my mother, brother and sister weekly in a Zoom call, I didn’t do that before’* (male, aged 60-64, responded before August 2021).

Participants who reported no reduction in contact with others during the pandemic often also reported low pre-pandemic levels of contact:

*‘I don’t like speaking to people but I never have’* (female, aged 70-74, responded after July 2021).

For others, pandemic-related changes of circumstances, such as moving in with a partner, increased contact:

‘*Speak to partner more as staying with him*’ (female, aged 75-79, responded before July 2021).

### Changes to Activities (n = 545)

Only 84/545 (15%) of respondents reported that the COVID-19 pandemic restrictions had no impact on their daily activities. For respondents who experienced change, activities outside of their home reduced:

*‘Yea our activities went to nothing, all our clubs and activities closed, even church, we didn’t really do anything’* (female, aged 75-79, responded after July 2021).

Some participants gave additional information as to which activities were affected, citing travel or holidays (n = 30, 5.5%), theatre outings (n = 25, 4.6%), going to the gym (n = 25, 4.6%), and in-person shopping (n = 24, 4.4%):

*‘I stopped going to pretty much all local shops, normally I would go two or three times a week and to the supermarket. I still go to the bakers and butchers but I’m restricted in that way’* (male, aged 65-69, responded before July 2021).

122 (22.4%) reported a reduction in one or more forms of exercise:

*‘Prior to lockdown I was going to the gym 3 times a week, and walking more. I had to shield over lockdown’* (male, aged 75-79, responded after July 2021).

Home based activities increased due to COVID-19 pandemic restrictions. For example, 31 (5.7%) engaged in more online activities; 26 (4.8%) performed more indoor activities and 17 (3.1%) did more gardening. One in 5 respondents (n = 87, 16%) walked more during the pandemic.

*‘I deliberately was going out for a walk, I was trying to walk more’* (female, aged 70-74, responded after July 2021).

These necessary changes to daily activities were often experienced negatively, even when overall activity levels were maintained:

*‘Before this all started, I was making improvements in going out more, felt more alive. I always struggled with anxiety, so this has always been a problem for me. When the pandemic began, I felt much more isolated. I was staying inside and playing board games with my brother’* (male, aged 70-74, responded after July 2021).

### Changes to Physical Wellbeing (n = 545)

Just over half of the older adults who answered this question (n = 275, 50.5%) reported no change to physical wellbeing; as shown in Table 2, reports of decline (in physical health, in fitness or exercise levels) were prominent in other narratives:

*‘I feel more stiff, more pains’* (female, aged 75-79, responded before July 2021).

8 (1.5%) of participants reported reduced access to healthcare services; this was described by a participant who had:

*‘issues with [their] hands but [it is] hard to see [a] GP about it so [they had] a delayed diagnosis and treatment’* (male, aged 80-84, responded after July 2021).

22 (4%) described weight gain:

*‘I put on a bit of weight because I’m not as active’* (male, aged 65-69, responded after July 2021).

A small number gave responses that we classified as improvements in physical health (n = 45, 8.3%), and/or an increase in exercise or fitness (n = 35, 6.4%):

*‘Yes – because he was doing his exercises every day and so he was fitter than he had been in a long time’* (male, aged 85-89, responded after July 2021).

The role of socioeconomic factors in enabling resilience was evident in this next response:

‘*We have a large garden, so there was plenty of gardening to do and because I have a workshop, and I am a “car nerd” I usually spend all my time there in the workshop, so luckily covid didn’t upset me because I could still do most of the things I enjoy doing*’ (male, aged 80-84, responded after July 2021).

For another participant, financial stresses made worse by the loss of employment negatively impacted sleep and wellbeing:

*‘Financial worry does keep me awake - like everybody I’m sometimes overwhelmed with the gravity of it’* (female and aged 64.4 years, responded after July 2021)

The loss of many routine medical services impacted physical wellbeing. In this next quote, a participant describes how a regular procedure that prevented a need for a catheter and incontinence was not available for a time in the pandemic:

‘*Doctors were closed down - my botox injections in my bladder stopped and I had a catheter installed, which I did not get on with it. I also went to a&e with my incontinence problems and they could not help either. It was hard*’ (male, aged 70-74, responded after July 2021).

### Changes to Diet (n = 545)

A third of respondents (n = 360, 66.1%) reported a change in diet.

26 (4.8%) of those that experienced change reported more healthy eating; and/or cooking at home more (n = 57, 10.5%):

*‘Before COVID, I used to go out to restaurants, now I cook my own food and I think I eat healthier, more fruits and veggies’* (male, aged 60-64, responded after July 2021).

31 (5.7%) ate more unhealthy foods including more chocolate (n = 8), cake (n = 8), biscuits (n = 4), and processed food (n = 5). Participants discussed these unhealthier eating habits:

*‘Never used to snack, now snacks more out of boredom/ being indoors’* (female, aged 65-69, responded before July 2021).

*‘I ate more sweets and chocolate than I should have’* (female, aged 75-79, responded after July 2021).

21 (3.9%) increased their overall food intake and 11 (2.0%) snacked more. One respondent described eating less, linking this to a loss of routine and confusion. Their response indicated a potential role of cognitive impairment as a barrier to the resilience evident in other responses, where routines were adapted, for example in this next quote:

*‘I think I eat less, I don’t know what to eat sometimes, I feel confusion around eating because I am home all day’* (female, aged 70-74, responded after July 2021).

One respondent explained how they managed to maintain weight loss despite not having access to their support group:

*‘I was really good during lockdown, I was in the Slimming World group but I couldn’t go, I managed to stay the same, I was stable on my weight, I didn’t lose but I kept it under control’* (female, 60-64, responded after July 2021).

### Changes to Mental Wellbeing (n = 540)

Nearly half of participants (48%, n = 257) described (almost exclusively negative) changes in their mental health due to the pandemic. The most frequently reported issues were lower mood (n = 220, 40.7%), increased worry or stress (n = 65, 12%), heightened anxiety (n = 33, 6.1%), fear of the virus (n = 24, 4.4%), and depression (n = 22, 4.1%). The decline in mental wellbeing can be seen in the following response:

*‘Since COVID started I’ve been put on antidepressants and the dosage has increased during the last 6 months. It’s the stress of COVID . . . My anxiety and depression has got a lot worse during COVID’* (female, aged 60-64, responded before July 2021).

22 (4.1%) of participants mentioned feeling more lonely when asked about their mental health. This next quote indicates how anxiety and isolation could be mutually reinforcing, creating a vicious cycle:

*‘I feel lonely, I’m scared, it’s changed a lot - when you sit in a room and can’t go out because you think you might catch it, I feel down, mentally keep thinking I won’t live any longer’* (male, aged 75-79, responded before July 2021).

Only 13 (2.4%) who experienced change reported improved mental health during the pandemic:

*‘I think on the whole I felt happier as I had more time to myself and time to think’* (male, aged 75-79, responded after July 2021).

### Changes to Practical Support (n = 546)

Just over half (56.4%, n = 308) of participants reported experiencing no change in how much they accessed practical support. Other responses described having less access to support from tradespeople and different means of accessing medical services (Table 2).

*‘[I] haven’t called in many professionals (gas service, cleaner etc.) so reduced the practical help that I would have wanted like repairs’* (male, aged 65-69, responded before July 2021).

For those who reported receiving more practical support, this was from family and friends who, for example, delivered shopping or meals.

*‘I’ve required more practical help during COVID e.g. my friends and family help me with shopping now as I’m classed as vulnerable’* (female, aged 60-64, responded before July 2021).

Many participants mentioned using more online shopping, an option that would not have been available to those on lower incomes. For others, there was a need to rely on ‘food parcels’ (male, aged 60-64, responded after July 2021), or friends and family. Some commented on the frustrations of this necessary reliance and increased dependency:

*‘Yes, had to rely on daughter bring in me food, so whatever she would get from the supermarket and I prefer to do it myself’* (female, aged 80-84, responded after July 2021).

### Changes to Emotional Support (n = 540)

A fifth of participants (22%; n = 119) experienced a change in how much emotional support they received; other respondents (n = 42) indicated they had less access to emotional support during the pandemic.

One participant, whose response was recorded verbatim by the researcher in an interview completed by video-call, described greater support needs but receiving less support:

*‘She felt really desperate and that’s why she called the IAPT [Improving Access to Psychological Therapies] service but no response. She didn’t get any help. She feels horrible and angry most of the time’* (female, aged 65-69, responded before July 2021).

Others (n = 35) reported a change to how they accessed emotional support with less face-to-face support. For example, 1 participant reported continuing support from: *‘The GP, but the way I was seeing the GP changed (virtually)’* (male, aged 70-74, responded after July 2021).

Remote connections did not always compensate for in-person contact. For some, changes in GP accessibility felt less supportive:

*‘I feel like I am struggling to get to the doctors, I am struggling to get help. It feels like doctors care less, just prescribing’* (female, aged 70-74, responded after July 2021).

Several participants described missing physical contact:

‘*I’m not able to hug my children and grandchildren*’ (male, aged 75-79, responded before July 2021).

Some (n = 17) participants reported needing and receiving more emotional support; emotional and practical support was often provided by neighbours:

*‘I’ve discovered how brilliant my neighbours are, they’re 6 different neighbours and they check up on me and bring me cooked food’* (female, aged 70-74, responded before July 2021).

### Changes to Provision of Support to Others (n = 538)

Around two-thirds 66.9% (n = 360) of respondents felt that COVID had not changed the support they provided to others; others (n = 95) provided more care, and/or more emotional support for friends and family (n = 13) or brought shopping or provisions to others (n = 16):

*‘The old lady below us – I’m more conscious to cook food for her to take down’* (female, aged 65-69, responded before July 2021).

Others (n = 59) were unable to provide as much support to others as they used to, mainly because they were unable to have contact with grandchildren or children they usually provided care for:

*‘Yes I have a daughter in a care home so haven’t seen her and haven’t been able to provide care for her’* (female, aged 65-69, responded before July 2021).

## Discussion

This is, to our knowledge, the largest survey asking older people living with mild cognitive concerns how government imposed COVID-19-related restrictions affected their lifestyle and wellbeing. Unsurprisingly, most respondents saw fewer people and were less active. Many participants adapted their daily routines, substituting activities that were no longer possible in the pandemic with home cooking, walking, gardening or online activities. Reports of reduced physical wellbeing, attributed to lower activity and exercise levels and reduced mental wellbeing (eg, low mood and anxiety), related to anxieties around the virus and loneliness were common.

Between the stressor (pandemic lockdowns and fears) and accounts of adaptation and accommodating to new contexts, lies ‘the resilience itself’.^17^ Social, digital, and community capital facilitated resilience. Most participants did not report any changes to the quantity of practical and emotional support they received, though systems for obtaining these changed. Some respondents compensated for less in-person contact by adopting video-call technology and using the phone more to connect with friends, families and health professionals. A minority spoke of the distress of isolation, lost opportunities to connect outside the home and reduced access to routine health care, indicating that while stoical accounts of adaptation and resilience predominated, this was not always possible.

One account indicated that greater cognitive impairment (confusion around new routines) may have impeded resilience. Social and digital exclusion affect those experiencing socioeconomic deprivation more, as financial resources facilitate access to transport, digital connectivity, health, community and local social services.^31^ While we did not measure socioeconomic barriers to resilience, or indeed resilience directly, they were suggested by some responses. Examples include where having a garden and a shed for hobbies supported resilience, and another where financial insecurity caused worry and insomnia. Online shopping helped many, while for others (who may have not had access to this for reasons including limited financial resources), greater reliance on friends, family or food parcels from community organisations brought sometimes unwelcome reliance on others. Concerns about physical deterioration due to reduced availability of routine care reflect reports of people living with long term conditions during the pandemic.^32^

Though older people with mild cognitive concerns are often highly motivated to reduce dementia risk, they are less likely to be able to do so successfully without support.^33^ One respondent described how she had managed to maintain, but not progress her goal for weight reduction without the support of her slimming group, which closed in the pandemic. Her account illustrated the importance of social support in achieving lifestyle change. By increasing reliance on remote connectivity, the pandemic reinforced effects of digital exclusion. The 2025 UK digital switchover will eliminate landlines, lifelines for many older people in the last pandemic,^34^ so this will be a pertinent issue in future preparedness.

### Public Health and Policy Implications

Global pandemics such as COVID-19 are relatively rare, but research has predicted an increased frequency in future.^35^ Despite UK government investment in infrastructure and economic growth to aid post-pandemic recovery, there has been little discussion of strategies to address pandemic-related health repercussions (46). The UK COVID-19 Public Inquiry Module 1, covering Resilience and Preparedness, concluded that Emergency planning failed to sufficiently consider health and social inequalities, and local authorities and volunteers were not adequately engaged.^36^ The ongoing Darzi independent investigation into the state of the NHS, which will inform a new NHS 10 year plan^37^ is an opportunity to focus on the prevention of dementia. This needs to include population-wide, primary prevention and public health messaging and interventions targeting at risk groups, including those with memory concerns.

While the particular challenges for people living with dementia were considered in the pandemic, perspectives and needs of people with mild cognitive impairments were not specifically acknowledged.^38^ Levels of psychological resilience and emotional wellbeing of people with mild cognitive concerns appear to be greater^39-41^ than for people living with dementia^8^; but lower than for older people without cognitive concerns who have reported good social support and drawing on previous coping strategies and life experience throughout the first wave of the pandemic.^42^

Future preparedness strategies should actively consider those living with mild cognitive concerns, who are often excluded from health and care planning; but whose resilience can be enhanced by community and digital connectivity. Ensuring that the population can be resilient to change may mean integrating online options (and providing skills training where needed) and in-person activities so any future shifts in the form of engagement are less disruptive to the routines of vulnerable groups. Creating community support networks where cognitively frail people are more digitally and socially connected will support resilience of this group and contribute to dementia prevention, now and in any future pandemic. Such work should account for intersectionality – the challenges people from more deprived socioeconomic groups who also have memory concerns may experience accessing such resources. Even since these interviews took place, more cognitively frail older people will have digital access, due to the twin impacts of societal digitalisation, expedited by the pandemic and cohort effects, as older generations increasingly have experience of internet use from earlier life. National prevention policies need to be cross-disciplinary, considering how to implement growing evidence that people with cognitive concerns can be supported to reduce their dementia risk,^43^ and ensure potentially vulnerable citizens are protected while online.^44,45^

### Limitations

Researchers summarised participants’ responses, which may have introduced bias. However, using the manifest analysis method, responses were coded based on what was said rather than interpreted. Although the survey asked specifically about recent changes due to COVID, level of restrictions varied over the study period, so respondents would have been experiencing these differently depending on when they completed it. We did not conduct a pilot study of the questionnaire, though it was piloted with a PPI member. We reviewed the questions and responses after the first 10 responses. The questions appeared to be well understood and acceptable. The lack of external validation limits interpretation of findings.

Respondents are unlikely to be a representative sample of the older population living with memory loss, as trial populations are not representative (48). A criterion for participation in the APPLE-Tree trial was willingness to engage in a video-call intervention to improve cognitive wellbeing, thus introducing a bias in favour of those with more awareness of the cognitive challenges they were experiencing. Devices were available for loan, but nonetheless those with their own devices, accustomed to online communication, were probably more likely to take part, as were those more amenable to group participation. A telephone interview study of older adults with MCI or SCD in Italy, reported fewer ongoing social activities, perhaps reflecting a greater proportion of people who were digitally excluded in their sample population, or differences in the level of pandemic social restrictions between countries.^39^ Despite these caveats, likely to have biased our sample towards greater resilience (through more socioeconomic, technology, and community capital), we think our findings can inform strategies for secondary dementia prevention, including preparedness for future global disasters. While we consider how technology and social connections increased resilience and discuss evidence that these barriers are often socioeconomically determined, we did not directly compare reports of lifestyle changes against socioeconomic characteristics in this descriptive study.

## Conclusion

We describe the accounts of people living with mild memory loss regarding how their wellbeing and lifestyle were influenced by the COVID pandemic. These evidenced increased loneliness, isolation and physical and mental distress, but also resilience, through moving to online social connections and adapting new daily activities. Our findings may inform how we protect people with mild cognitive concerns in future disasters. Reducing the digital divide and tackling loneliness in older people now could reap dividends, including in any future pandemic.

## Supplemental Material

sj-pdf-1-inq-10.1177_00469580251332062 – Supplemental material for Social, Digital and Community Capital Facilitated COVID-19 Pandemic Resilience in a Qualitative Survey of Older Adults With Mild Cognitive Concerns Supplemental material, sj-pdf-1-inq-10.1177_00469580251332062 for Social, Digital and Community Capital Facilitated COVID-19 Pandemic Resilience in a Qualitative Survey of Older Adults With Mild Cognitive Concerns by Annie Mae Wright, Harriet Demnitz-King, Alexandra Burton, Rachel M. Morse, Sweedal Alberts, Charlotte Kenten, Rosario Isabel Espinoza Jeraldo, Michaela Poppe, Julie Barber and Claudia Cooper in INQUIRY: The Journal of Health Care Organization, Provision, and Financing

## Appendix 1

### Coding Tree

Changes in who you speak to each week

**Table:**

Key			
N = No change			
= No answer/didn’t answer question			
SP = Same people			
Online = More online calling			
Phone = More phone calling			
LI = Less in-person			
LP = Less people			
MP = More people			
L/M = Some people more, some people less			
LO = Less often			
MO = More often			
I = Feel more isolated			

Changes in what you eat

**Table:**

**Key**			
N	no change		
	no answer/didn’t answer question		
HE	healthy eating		
CO	cooked at home more		
RE	ate out less		
MF	more food		
MS	more snacks		
UN	more unhealthy food		
CH	chocolate		
CA	cake		
RM	ready meals/processed food		
BI	biscuits		
C	other change		

Changes in what activities you do

**Table:**

**Key**			
N	no change		
	no answer/didn’t answer question		
LA	reduction in one or more activities		
LS	less socialising		
LE	less exercise		
G	no gym		
TH	no theatre		
L	no library		
CC	no community centre		
S	no shopping		
M	no music/concerts		
F	no football matches		
TR	no travelling/holidays		
O	more online activities		
IA	more indoor activities		
ME	more exercise		
MW	more walking		
R	found alternative exercise		
MG	more gardening		

Practical help

**Table:**

**Key**			
N	no change		
	no answer/didn’t answer question		
LS	less access to practical help		
MS	had/needed more support		
D	didn’t need practical help		
SS	had help with shopping/food		
NC	restricted access to cleaners/tradesmen/gardeners		
GP	difficulty accessing medical services		
S	had support from family/friends		
EX	had help from external services		

Emotional support

**Table:**

**Key**			
N	no change		
	no answer/didn’t answer question		
LS	less access to EMOTIONAL? help		
MS	had/needed more support		
D	didn’t need practical help		
SS	had help with shopping/food		
NC	restricted access to cleaners/tradesmen/gardeners		
GP	difficulty accessing medical services		
S	had support from family/friends		
EX	had help from external services		

Mental wellbeing

**Table:**

**Key**			
N	no change		
	no answer/didn’t answer question		
?	some change, don’t specify type of change		
WM	worse mood		
BM	better mood		
W	more worried/stressed		
F	fear of COVID		
A	more anxious		
FR	frustrated		
AN	angry		
B	more bored		
D	more depressed		
L	isolated/lonely		
I	more irritable/moody		
WS	worse sleep		

Physical wellbeing

**Table:**

**Key**			
N	no change		
	no answer/didn’t answer question		
?	some change, don’t specify type of change		
BP	better physical health		
WP	worse physical health		
A	increased aches/pains		
MS	reduced access to medical services		
WG	weight gain		
ME	more exercise		
RE	reduced exercise		

Providing support

**Table:**

**Key**			
N	no change		
	no answer/didn’t answer question		
P	providing more care		
L	unable to provide as much care		
D	don’t care for anyone		
E	providing more emotional support		
S	bringing provisions to others		
B	cannot provide childcare		

## Appendix 2

### Consolidated criteria for reporting qualitative studies (COREQ): 32-item checklist

#### Domain 1: Research Team and Reflexivity

##### Personal Characteristics

1. Interviewer/facilitator Which author/s conducted the interview or focus group?: Methods, procedures section (page 6)
2. Credentials What were the researcher’s credentials? For example, PhD, MD: Methods, procedures section (page 6)
3. Occupation What was their occupation at the time of the study?: Methods, procedures section (page 6)
4. Gender Was the researcher male or female?: Methods, procedures section (page 6)
5. Experience and training What experience or training did the researcher have? Methods, procedures section (page 6)

##### Relationship With Participants

6. Relationship established Was a relationship established prior to study commencement? Methods, procedures section (page 6)
7. Participant knowledge of the interviewer: What did the participants know about the researcher? For example, personal goals, reasons for doing the research. Methods, procedures section (page 6)
8. Interviewer characteristics What characteristics were reported about the interviewer/ facilitator? For example, Bias, assumptions, reasons and interests in the research topic

#### Domain 2: Study Design

##### Theoretical Framework

9. Methodological orientation and Theory

What methodological orientation was stated to underpin the study? For example, grounded theory, discourse analysis, ethnography, phenomenology, content analysis: Methods, analysis section (page 6)

###### Participant Selection

10. Sampling How were participants selected? For example, purposive, convenience, consecutive, snowball: Methods, study sample, page 4
11. Method of approach How were participants approached? For example, face-to-face, telephone, mail, email: Methods, study sample, page 4
12. Sample size How many participants were in the study?: Results, sample description, page 6
13. Non-participation How many people refused to participate or dropped out? Reasons? Results, sample description, page 6/7

###### Setting

14. Setting of data collection Where was the data collected? For example, home, clinic, workplace: Methods: procedure page 5/6
15. Presence of non-participants Was anyone else present besides the participants and researchers? Methods: procedure page 5/6
16. Description of sample What are the important characteristics of the sample? For example, demographic data, date: Table 1

###### Data Collection

17. Interview guide Were questions, prompts, guides provided by the authors? Was it pilot tested? Methods, procedure, page 5
18. Repeat interviews Were repeat interviews carried out? If yes, how many? Methods, procedure, page 5/6
19. Audio/visual recording Did the research use audio or visual recording to collect the data? Methods, procedure, page 5/6
20. Field notes Were field notes made during and/or after the interview or focus group? Methods, procedure, page 5/6
21. Duration What was duration of the interviews or focus group? Methods, procedure, page 5/6
22. Data saturation Was data saturation discussed? No
23. Transcripts returned Were transcripts returned to participants for comment and/or correction? No

#### Domain 3: Analysis and Findings

##### Data Analysis

24. Number of data coders How many data coders coded the data? Two; Methods: data analysis, page 6
25. Description of the coding tree Did authors provide a description of the coding tree? Methods: data analysis, page 6
26. Derivation of themes Were themes identified in advance or derived from the data? Methods: data analysis, page 6
27. Software What software, if applicable, was used to manage the data? Methods: data analysis, page 6
28. Participant checking Did participants provide feedback on the findings? No
Reporting
29. Quotations presented Were participant quotations presented to illustrate the themes/findings? Was each quotation identified? For example, participant number yes – throughout results section
30. Data and findings consistent Was there consistency between the data presented and the findings? Yes, throughout results section
31. Clarity of major themes Were major themes clearly presented in the findings? yes
32. Clarity of minor themes Is there a description of diverse cases or discussion of minor themes? Yes
