# Supplementary material for: Social, Digital and Community Capital Facilitated COVID-19 Pandemic Resilience in a Qualitative Survey of Older Adults With Mild Cognitive Concerns
Source: Inquiry. 2025 Jun 13;62:00469580251332062. doi: 10.1177/00469580251332062 (PMC12171272; doi:10.1177/00469580251332062)
Supplement: sj-pdf-1-inq-10.1177_00469580251332062 – Supplemental material for Social, Digital and Community Capital Facilitated COVID-19 Pandemic Resilience in a Qualitative Survey of Older Adults With Mild Cognitive Concerns [file sj-pdf-1-inq-10.1177_00469580251332062.pdf]

APPLE-TREE STUDY	Site ID:		Participant initials:		
	Participant ID:		Date of assessment:	D D M M Y Y Y Y	

IMPACT OF SOCIAL CHANGES DUE TO COVID-19 QUESTIONNAIRE

1. Do you have access to (please tick all that apply):

   a. Mobile phone ☐

   b. Smart phone ☐

   c. Video calling on line (zoom or skype) (if so which) ☐

   ____

2. How did the recent changes due to Covid change: [prompt for during isolation / after]

   a. Who you speak to each week

   ____
   ____
   ____
   ____

   b. What you eat

   ____
   ____
   ____
   ____

c. What activities you do

____

____

____

____

d. How you access help and who you turn to if you need

(1) emotional support ________________________________

____

____

____

(2) practical help ________________________________

____

____

____

e. Your mental wellbeing (prompt for worries, mood)

____

____

____

____

f. Your physical wellbeing

____

____

____

____

g. Who you provide care for

____

____

____

____
